# Magnitude and associated factors of repeat induced abortion among reproductive age group women who seeks abortion Care Services at Marie Stopes International Ethiopia Clinics in Addis Ababa, Ethiopia

**DOI:** 10.1186/s12978-019-0743-4

**Published:** 2019-06-04

**Authors:** Bethelihem Alemayehu, Adamu Addissie, Wondimu Ayele, Sisay Tiroro, Demelash Woldeyohannes

**Affiliations:** 1 Marie Stopes International Ethiopia Clinic, Addis Ababa, Ethiopia; 2 School of Public Health, College of Health Sciences, Department of Preventive Medicine, Addis Ababa University, Addis Ababa, Ethiopia; 3 National Defense Health Main Department, Health promotion and Disease prevention department, Addis Ababa, Ethiopia; 4 Department of Public Health, Collage of Medicine and Health Science, Madda Walabu University, Bale Goba, Ethiopia

**Keywords:** Repeat induced abortion, Reproductive age group, Ethiopia

## Abstract

**Background:**

Repeated induced abortion is important public health concern both in the developing and developed world that increases maternal morbidity and mortality. The aim of this study was to determine the magnitude and associated factors of repeated induced abortion among abortion care service seekers at Marie Stopes International Ethiopia clinics in Addis Ababa, Ethiopia.

**Methods:**

A cross sectional study was conducted among 429 women seeking abortion care at Marie Stopes International Ethiopia clinics. Simple random sampling technique was used to select study participants. Data were collected by trained data collectors using pretested structured questionnaires. Data were checked for completeness, consistency, coded and entered and analyzed through SPSS version 20. Bivariate and multivariate logistic regression analysis was computed to test the strength of association and the *p*-value < 0.05 was considered as statistical significant.

**Result:**

The magnitude of repeat induced abortion was 33.6%. Based on this study age groups 20–24 years (AOR = 1.2; CI: 1.1–2.3), 25–29 years (AOR = 5.4; CI: 3.1–6.2) and 30–34 years (AOR = 1.1; CI: 1.02–2.6); respondents with the educational level of primary (AOR = 0.2; CI: 0.070.6), secondary (AOR = 0.4; CI: 0.2–0.8) and college diploma and above (AOR = 0.4; CI: 0.2–0.6); those with the monthly income of 1001–2000 Ethiopian birr (AOR = 4.2; CI: 1.8–9.4) and 2001–3000 Ethiopian birr (AOR = 0.3; CI: 0.2–0.9); those with years in marriage with 1–2 years (AOR = 2.4; CI: 1.2–4.9) and those with last time of abortions of 1–2 years, 2–3 years and above 3 years, (AOR = 0.2; CI: 0.1–0.5), (AOR = 0.1; CI: 0.05–0.4), (AOR = 0.4; CI: 0.2–0.9), respectively were found to be significantly associated with repeat induced abortions.

**Conclusion and recommendation:**

The magnitude of repeat induced abortion is similar with the reports from developing countries but it was lower than that of developed countries. Age group (20–24, 25–29 and 30–34 were positively associated with repeat induced abortion), educational level (primary, secondary and collage diploma and above were negatively associated with repeat induced abortion), monthly income (earn 1001–2000 Ethiopian birr were positively where as monthly income between 2001 and 3000 Ethiopian birr negatively associated), years in marriage (1–2 years was negatively associated) and time of last abortion (1–2 years, 2-3 years and above the three years were negatively associated) were the associated factors for repeat induced abortion. Health promotion messages are needed to focus to improve the knowledge of women about contraceptives as a primary prevention of repeated induced abortion.

## Plain English summary

Estimated 42 million induced abortions each year, nearly 20 million are performed in unsafe conditions and/or by unskilled providers and result in the deaths of an estimated 47,000 girls and women. This represents about 13% of all pregnancy-related deaths. Almost all unsafe abortions take place in developing countries, and this is where 98% of abortion-related deaths occur. This study aimed in showing the magnitude and associated factors of repeat induced abortion at facility based data. This will help for the policy makers and program implementers to consider the prevention of repeat induced abortion and its associated factors at facility and community based level. Facility based cross-sectional study design was conducted to assess the magnitude and associated factors of repeat induced abortion among the women who seeks abortion services at Marie Stopes International Ethiopia Clinics in Addis Ababa. From 442 95% of the study participants were included in the study. The mean age of the study participants was 26 years. This study showed the magnitude of repeat induced abortion was 33.6%. Whereas study participants age groups, respondents educational level, monthly income, years in marriage and the time for last abortions was the factors associated with induced abortion. In conclusion the magnitude of repeat induced abortion is similar with the reports from developing countries but it was lower than that of developed countries. Hence, health promotion messages are needed to focus to improve the knowledge of women about contraceptives as a primary prevention of repeated induced abortion.

## Introduction

Abortion is a sensitive and contentious issue with religious, moral, cultural, and political dimensions. It is also a public health concern in many parts of the world. The World Health Organization (WHO) estimates that worldwide 210 million women become pregnant each year and that about two-thirds of them, or approximately 130 million, deliver live infants. The remaining one third of pregnancies ends in miscarriage, stillbirth, or induced abortion. Of the estimated 42 million induced abortions each year, nearly 20 million are performed in unsafe conditions and/or by unskilled providers and result in the deaths of an estimated 47,000 girls and women. This represents about 13% of all pregnancy-related deaths. Almost all unsafe abortions take place in developing countries, and this is where 98% of abortion-related deaths occur [1].

A studies shows unsafe abortions were responsible for nearly one-third of maternal deaths in Africa, and WHO reports that in the countries of sub-Saharan Africa unsafe abortions are responsible for 50% of maternal deaths. Women in developed and developing regions of the world turn to abortion at similar rates; annually, 29 abortions are performed per 1000 women in developing countries, compared with 26 per 1000 women in developed countries [1, 2].

The ages at which women have repeat induced abortion differ markedly across regions. Nearly 60% of women in sub-Saharan Africa who have repeat induced abortion are younger than 25, and 25% are still in their teens. In Asia, 70% of repeat induced abortions are among women 25 and older; many of them already have children and want to limit family size. In Latin America and the Caribbean, more than half of repeat induced abortions occur among women who are in their 20s, suggesting that women in this region use repeat induced abortion to space births and limit family size [3].

Furthermore, there is no standard definition of repeat induced abortion; some studies count more than one abortion ever, others focused on multiple abortions within shorter intervals [4, 5]. A Canadian study found more similarities than differences among first-time and repeat abortion clients [6]. More recent research has identified an array of potential risk factors for repeat induced abortion including: age; socioeconomic status; parity; education; foreign origin; race; smoking; alcohol/drug abuse; physical abuse or violence; early sexual debut; previous contraceptive use; and type of contraceptives used [5].

Previous research mainly in high income countries has identified common risk factors for repeat abortions, including higher age, higher parity, and lower socioeconomic status. Evidence from the United States clearly showed that the women with repeat abortion were as highly as those with one -time abortion to use contraception [6–8].

In Russia one clinic based survey estimated that repeat abortion accounts for approximately 60% of all abortions and another Russian community based study revealed that repeat abortions were associated with low education and alcohol use, and it is more common under 35 years of women [9, 10].

Facility based cross sectional study at Nepal indicated that the magnitude of repeat abortion among abortion service care seeker is 32.3% (95% CI 29.6–34.9) and this study determined that age and parity, and women with no intention of having a future child, with those attaining primary or secondary education level and those attending the non-governmental sector clinics were the main associated factors for repeat abortion among the abortion care seekers [11].

A facility based cross sectional study in Sweden outlined that parity, lack of emotional support, being unemployed or on sick leave, daily tobacco use, and compulsory school or high school as the highest educational level were the associated factors for repeat abortion among women aged 20–49 years [12].

Different studies showed that various risk factors for repeat abortion, for instance, the risk of repeat abortion has been found to be related to several socioeconomic factors, such as immigrant status and weak social networks [12, 13], low educational level and unemployment [14, 15]. A correlation between repeat abortion and a history of violence and sexual abuse has been found [16], and parity and smoking are more common among women with repeat abortions [17, 18].

Even some studies done some years back in Ethiopia, which shows the magnitude and associated factors for induced abortion at community and facility base, but still there is limitation of studies done on the magnitude and associated factors for repeat induced abortion. So, this study aimed in showing the magnitude and associated factors of repeat induced abortion at facility based data. This will help for the policy makers and program implementers to consider the prevention of repeat induced abortion and its associated factors at facility and community based level.

## Methodology

### Study area and period

The study was conducted at Marie Stopes International Ethiopia (MSIE) clinics in Addis Ababa, Ethiopia which is non-governmental profit based organization providing different kinds of services to the community, including FP services, ANC, PNC, deliver, abortion and post abortion care services in large. Before one month of starting this study a total of 1209 women received abortion services in all branches of MSIE clinics (i.e. Kirkose 399, Arada 360, Teklehaymenot 279 and Gulale 180) in Addis Ababa. This study was conducted from April 1 to April 30, 2015 at MSIE clinics in Addis Ababa, Ethiopia.

### Study design

Facility based cross-sectional study design was conducted to assess the magnitude and associated factors of repeat induced abortion among the women who seeks abortion services at MSIE clinics in Addis Ababa, Ethiopia.

### Population

#### Source population

The source populations for this study were all women who were (at the age of 15–49 years) taken abortion care services of the MSIE clinics in Addis Ababa, Ethiopia.

#### Study population

All women aged 15–49 who were seeking abortion care services during the study period.

### Inclusion and exclusion criteria

#### Inclusion criteria

All women aged 15–49 who were received abortion care services from MSIE clinics were included in the study.

#### Exclusion criteria

Women who were not volunteer to participate in the study, women who were critically ill and women who were not available during the data collection period were excluded from the study.

### Sample size determination

The sample size of the study was determined using the single population proportion formula based on the assumption that the prevalence rate of repeat induced abortion was 50% (since there is no previous studies done on the area), and 95% confidence interval was used with a marginal error of 5% and by taking the non-response rate as 15% due to sensitivity of the issue.

$$ \mathrm{n}=\frac{{\left({\mathrm{Z}}_{\alpha /2}\right)}^2\times \mathrm{P}\ \left(1-\mathrm{P}\right)}{{\mathrm{d}}^2} $$

Where: n = sample size.

P = proportion of repeat induced abortion.

q = 1-p.

d = desired degree of precision (5%).

Z = is the standard normal value at 95% confidence level, which is 1.96

$$ \mathrm{n}=\frac{(1.96)^2\times 0.5\times 0.5}{(0.05)^2}=384.16=15\%\mathrm{of}\ \mathrm{n}\mathrm{on}\hbox{-} \mathrm{response}\ \mathrm{rate}\ \mathrm{was}\ \mathrm{assumed},\mathrm{the}\ \mathrm{total}\ \mathrm{sample}\ \mathrm{size}\ \mathrm{was}\ 442 $$

### Sampling procedure

For each Marie Stopes service provision unit the allocated sample size was calculated using the monthly total number of women of all the service provision units, the monthly visiting number women from each unit and the total sample size. Finally simple random sampling methods were employed to select the study participants from each service provision units of MSIE clinics.

### Study variables

#### Dependent variable

Repeat induced abortion.

#### Independent variables

Socio-demographic characteristics (age, marital status, educational status, economic status, residence), Years in marriage, Family history, Number of pregnancy, Time of last pregnancy, Number of previous abortion, Time of last abortion, and Methods used for last abortion.

### Operational definition

**Repeat induced abortion**: - refers for the women who having more than one pregnancy termination, without any medical or surgical indication.

### Data collection tools and procedures

The data collection instruments were structured questionnaires which consist of both open and closed ended questions. It was first prepared in English version and latter on translated to Amharic version, which is local language. The data collectors were eight diploma nurses who were working outside of MSIE clinics, and additional three BSc nurses were recruited for supervision. Full 3 days training was given for data collectors and supervisors on the methods of collecting data through interviewing the clients. How to fill the information on questionnaire, the ethical aspect in keeping the confidentiality of their information were another focus of the training. The supervisors had monitored the data collection process of the data collectors and taken corrective measures with consultation of principal investigator.

### Data quality control

To ensure the quality of the data, the questionnaire was prepared using simple and easily understandable language and also translated in to local language (Amharic), and 3 days training was given for data collectors prior to data collection. Pre-test was done outside the study area on 5% questionnaires and modifications were done accordingly. During data collection, the supervisors monitored the collection process by checking completeness of the data and took the correction on the spot of data collection site when any problem happened and the principal investigator rechecked the completeness of the data. Data were checked again for its completeness during data entry and the cleaning process was done by running simple frequency after data entry for its consistency. When inconsistency happens to the data, it was checked again by referring the hard questionnaire. Finally, data analysis was begun after completion of the cleaning process.

### Data processing and analysis

Data was collected from the respondents; entry and analysis were done using SPSS, version 20. Descriptive statistics such as frequency, proportion, means and standard deviation (SD) were computed. To estimate the magnitude of the association between associated factors and repeat induced abortion, odds ratio (OR) with 95% confidence intervals (CIs) was used. A logistic regression model was used for both bivariate and multivariate analysis in order to identify associated factors of repeat induced abortion among groups of independent variables. Variables which were significantly associated (*p* <  0.05) with repeat induced abortion in the binary logistic regression analysis were entered in to the multivariate logistic regression analysis model. The findings were expressed in AOR with 95% CIs and significant threshold was declared at *p* <  0.05.

### Ethical consideration

This study was done after getting ethical clearance from Research and ethical committee of the school of public health, Addis Ababa University. Written permission was also secured from MSIE clinics and verbal consent was obtained from the participants during the data collection.

## Result

### Socio-demographic characteristics of the study participants

A total of 429 participants with a mean age of 26 years were included in this study with a response rate was 97% (i.e. 30 study participants were not voluntary to participate on this study), of these 41% of them were at the age interval of 25–29 years. 91% of the study participants were from urban areas. Regarding to marital status 55% of them married and 37.5% were never married **(**Table 1**).**

**Table 1:** The socio-demographic characteristics of the women who seek abortion care services at MSIE clinics in Addis Ababa, Ethiopia, from April 1 to April 30, 2015

Variables	Number (*n* = 429)	Percent
Age		
15–19	20	4.7
20–24	133	31.0
25–29	176	41.0
30–34	88	20.5
35–39	12	2.8
Residence		
Urban	393	91.6
Rural	36	8.4
Educational status		
Illiterate	36	8.4
Elementary	92	21.4
Secondary	138	32.2
College diploma & above	163	38.0
Religion		
Orthodox	330	76.9
Muslim	71	16.6
Protestant	24	5.6
Others	4	0.9
Ethnicity		
Amhara	213	49.7
Oromo	88	20.5
Tigre	46	10.7
Gurage	66	15.4
Others	16	3.7
Marital status		
Married	236	55.0
Never married	161	37.5
Divorced	28	6.5
Widowed	4	0.9
Occupational status		
House wife	83	19.3
Student	39	9.1
Government employee	46	10.7
Private employee	233	52.0
House made	24	5.6
Others	14	3.3
Monthly income (ETB)		
< 500	149	34.7
501–1000	67	15.6
1001–2000	59	13.8
2001–3000	44	10.3
> 3001	110	25.6

ETB Ethiopian Birr, < = less than, > = more than

### Past and current reproductive history of the study participants

Of the total respondents, 268 of them had past and current reproductive histories and 41% had married at the age of 15–19 years and 29 % of them were in marriage for less than 1 year. Medications were used by 66% of the respondents to induce abortion **(**Table 2**)**.

**Table 2:** Past and current reproductive history of the women who seek abortion care services at MSIE clinics in Addis Ababa, Ethiopia, from April 1 to April 30, 2015

Variable	Frequency	Percent
Age at marriage		
15–19	112	41.8
20–24	96	35.8
25–29	52	19.4
30–34	8	1.9
Total	268	100.0
Years in marriage		
≤ 1 year	128	29.8
1–2 years	59	13.8
2–5 years	73	17.0
5–10 years	81	18.9
≥ 10 years	88	20.5
Total	429	100.0
Family size		
1–2	140	32.6
3–4	152	35.4
5–6	105	24.5
≥ 7	32	7.5
Total	429	100.0
Number of pregnancy		
1	116	27.0
2	140	32.6
3	78	18.2
4	63	14.7
5	16	3.7
≥ 6	16	3.7
Total	429	100.0
Time of last pregnancy		
< 1 year	265	61.8
1–2 years	127	29.6
2–3 years	23	5.4
≥ 3 years	14	3.3
Total	429	100.0
Was the last pregnancy wanted		
Yes	119	27.7
No	310	72.3
Total	429	100.0
Age of the last pregnancy		
< 1 year	59	24.2
1–2 years	58	23.8
2–3 years	34	13.9
≥ 3 years	93	38.1
Total	244	100.0
Number of previous abortion		
1 time	285	66.4
> =2 times	144	33.6
Total	429	100.0
Reason of abortion		
Being single	135	35
Schooling	75	17.5
To space	16	3.7
Economic problem	144	33.6
Separation	39	9.1
Others	20	4.7
Total	429	100.0
Time of last abortion		
Below 1 year	245	57.1
1–2 years	92	21.4
2–3 years	41	9.6
Above 3 years	51	11.9
Total	429	100.0
Abortion was performed by		
Trained person	421	98.1
Untrained person	4	0.9
Self-abortion	4	0.9
Methods used for abortion		
Medications	283	66.0
MVA	146	34.0
Total	429	100.0

Note: MVA Manual Vacuum Aspiration

### Factors associated with repeat induced abortion at MSIE clinics

#### Socio-demographic characteristics associated with repeat induced abortion

On bivariate analysis age of the study participants, educational status and monthly income were significantly associated with the outcome variable, but residence, marital status and occupation were not associated with the outcome variable. Study participants who were at the educational level of diploma and above were 3 times (COR = 3.0, CI; 1.3–7.1) more likely to have repeat induced abortion than illiterates **(**Table 3**)**.

**Table 3:** Socio-demographic characteristics associated with repeat induced abortion among the women who seek abortion care services at MSIE clinics in Addis Ababa, Ethiopia, from April 1 to April 30, 2015

Variables	Induced abortion		COR,95% CI	*P*-value
	Aborted once *N* (%)	Repeat abortion *N* (%)		
Age				
15–19	20 (7.0)	0 (0.0)	0.00	0.998
20–24	89 (31.2)	44 (30.6)	0.2(.07–0.8)	0.029
25–29	120 (42.1)	56 (38.9)	0.2 (0.06–0.8)	0.022
30–34	52 (18.2)	36 (25.0)	0.3 (0.09–1.2)	0.102
35–39	4 (1.4)	8 (5.6)	1.0	
Residence				
Urban	261 (91.6)	132 (91.7)	1.0 (0.5–2.0)	0.975
Rural	24 (8.4)	12 (8.3)	1.0	
Educational status				
Illiterate	28 (9.8)	8 (5.6)	1.0	
Elementary	64 (22.5)	28 (19.4)	1.5 (0.6–3.7)	0.355
Secondary	106 (37.2)	32 (22.2)	1.1 (0.4–2.5)	0.902
College diploma & above	87 (30.5)	76 (52.8)	3.1 (1.3–7.1)	0.009
Marital status				
Married	152 (53.3)	84 (58.3)	1.0	
Single	113 (39.6)	48 (33.3)	0.7 (0.5–1.2)	0.231
Divorced	16 (5.6)	12 (8.3)	1.3 (0.6–3.0)	0.451
Widowed	4 (1.4)	4 (0.9)	0.000	0.999
Occupation				
House wife	55 (19.3)	28 (19.4)	1.0	
Student	27 (9.5)	12 (8.3)	0.8(.3–1.9)	0.745
Government employee	34 (11.9)	12 (8.3)	0.7 (0.3–1.5)	0.370
Private employee	143 (50.2)	80 (55.6)	1.1 (0.6–1.8)	0.728
House made	20 (7.0)	4 (2.8)	0.4 (0.1–1.2)	0.116
Others	6 (2.1)	8 (5.6)	2.6 (0.8–8.2)	0.101
Monthly income				
< 500	97 (34.0)	52 (36.1)	1.1 (0.6–1.8)	0.715
501–1000	55 (19.3)	12 (8.3)	0.4 (0.2–0.9)	0.034
1001–2000	27 (9.5)	32 (11.2)	2.4 (1.2–4.4)	0.007
2001–3000	32 (11.2)	12 (8.3)	0.7 (0.3–1.6)	0.510
≥ 3000	74 (26.0)	36 (25.0)	1.0	

### Past and current reproductive histories associated with repeat induced abortion at MSIE clinics

On bivariate analysis years in marriage and time of last pregnancy were significantly associated with repeat induced abortion, but family size, time of last pregnancy, planned pregnancy, reason of the abortion and methods used for the abortion were not associated with repeat induced abortion. Women who were 5–10 years in marriage were 2 times (COR = 2.3, CI; 1.3–4.2) more likely to have repeat induced abortion than those who were less than 1 year **(**Table 4**)**.

**Table 4:** Past and current reproductive histories associated with repeat induced abortion among the women who seeks abortion care services at MSIE clinics in Addis Ababa, Ethiopia, from April 1 to April 30, 2015

Variables	Induced abortion		COR,95%CI	*P*-value
	Aborted once *N* (%)	Repeat abortion *N* (%)		
Years in marriage				
Less than 1 year	92 (32.3)	36 (25.0)	1.0	
1–2 years	48 (16.8)	11 (7.6)	0.5 (0.2–1.2)	0.168
2–5 years	42 (14.7)	31 (21.5)	1.8 (1.0–3.4)	0.039
5–10 years	42 (14.7)	39 (27.1)	2.3 (1.3–4.2)	0.004
Above 10 years	61 (21.4)	27 (18.8)	1.1 (0.6–2.0)	0.685
Total	66.4%	33.6%		
Family size				
1–2	88 (30.9)	52 (36.1)	0.9 (0.4–2.1)	0.970
3–4	116 (40.7)	36 (25.0)	0.5 (0.2–1.1)	0.110
5–6	61 (21.4)	44 (30.6)	1.2 (0.5–2.7)	0.657
Above 7	20 (7.0)	12 (8.3)	1.0	
Time last of pregnancy				
< 1 year	161 (56.5)	104 (72.2)	1.6 (0.5–5.2)	0.428
1–2 years	97 (34.0)	30 (20.8)	0.7 (0.2–2.6)	0.682
2–3 years	17 (6.0)	6 (4.2)	0.8 (0.2–3.9)	0.869
Above 3 years	10 (3.5)	4 (2.8)	1.0	
Was the pregnancy planed				
Yes	79 (27.7)	40 (27.8)	1.0	
No	206 (72.3)	104 (72.2)	0.9 (0.6–1.5)	0.990
Reason of the abortion				
Being single	84 (29.5)	51 (35.4)	2.4 (0.7–7.6)	0.130
Schooling	50 (17.5)	25 (17.4)	2.0 (0.6–6.6)	0.256
Spacing	12 (4.2)	4 (2.8)	1.3 (0.2–6.4)	0.720
Economic problem	100 (35.1)	44 (30.6)	1.7 (0.5–5.50	0.336
Separation	23 (8.1)	16 (11.10	2.7 (0.7–9.8)	0.114
Others	16 (5.6)	4 (2.8)	1.0	
Time of last abortion				
1 month-1 year	149 (52.3)	96 (66.7)	1.0	
1–2 years	69 (24.2)	23 (16.0)	0.5 (0.3–0.8)	0.016
2–3 years	32 (11.2)	9 (6.2)	0.4 (0.2–0.9)	0.038
Above 3 years	35 (12.3)	16 (11.1)	0.7 (0.3–1.3)	0.297
Methods used for abortion				
Medication	183 (64.2)	100 (69.4)	1.0	
MVA	102 (35.8)	44 (30.6)	0.7 (0.5–1.2)	0.280

Note: *MVA* Manual Vacuum Aspiration

### Multivariate analysis for factors associated with repeat induced abortion at MSIE clinics

After controlling for potential confounder on multiple logistic regression analysis age, educational status, monthly income, years in marriage, time of last abortion were significantly associated with repeat induced abortion among abortion care seekers at MSIE clinics in Addis Ababa, Ethiopia. Respondents who were at the age of 25–29 years were 5.4 (AOR = 5.4, 95%CI; 3.1–6.2) times more likely to had repeat induced abortion than those who were at the age of 15–19 years. Study participants who were illiterates were 60% less risky than those who have secondary and college diploma educational levels **(**Table 5**)**.

**Table 5:** Multivariate analysis for factors associated with repeat induced abortion among women who seeks abortion care services at MSIE clinics in Addis Ababa, Ethiopia, from April 1 to April 30, 2015

Variables	Induced abortion		COR,95%CI	AOR,95%CI	*P*-value
	Aborted Once	Repeat Abortion			
Age					
15–19	20 (7.0)	0 (0.0)	0.00	0.00	0.00
20–24	89 (31.2)	44 (30.6)	0.2(.07–0.8)	1.2 (1.1–2.3)	0.001
25–29	120 (42.1)	56 (38.9)	0.2 (0.06–0.8)	5.4 (3.1–6.2)	0.001
30–34	52 (18.2)	36 (25.0)	0.3 (0.09–1.2)	1.1 (1.02–2.6)	0.009
35–39	4 (1.4)	8 (5.6)	1.0	1.0	
Educational status					
Illiterate	28 (9.8)	8 (5.6)	1.0	1.0	
Elementary	64 (22.5)	28 (19.4)	1.5 (0.6–3.7)	0.2 (0.07–0.6)	0.006
Secondary	106 (37.2)	32 (22.2)	1.1 (0.4–2.5)	0.4 (0.2–0.8)	0.011
College diploma & above	87 (30.5)	76 (52.8)	3.1 (1.3–7.1)	0.4 (0.2–0.6)	0.001
Monthly income					
< 500	97 (34.0)	52 (36.1)	1.1 (0.6–1.8)	1.5 (0.8–2.9)	0.205
501–1000	55 (19.3)	12 (8.3)	0.4 (0.2–0.9)	0.9 (0.36–2.2)	0.819
1001–2000	27 (9.5)	32 (11.2)	2.4 (1.2–4.4)	4.2 (1.8–9.4)	< 0.001
2001–3000	32 (11.2)	12 (8.3)	0.7 (0.3–1.6)	0.3 (0.2–0.9)	0.039
≥ 3000	74 (26.0)	36 (25.0)	1.0	1.0	
Years in marriage					
Less than 1 year	92 (32.3)	36 (25.0)	1.0	1.0	
1–2 years	48 (16.8)	11 (7.6)	0.5 (0.2–1.2)	2.4 (1.2–4.9)	0.047
2–5 years	42 (14.7)	31 (21.5)	1.8 (1.0–3.4)	1.8 (0.8–3.9)	0.139
5–10 years	42 (14.7)	39 (27.1)	2.3 (1.3–4.2)	1.1 (0.5–2.8)	0.689
Above 10 years	61 (21.4)	27 (18.8)	1.1 (0.6–2.0)	0.7 (0.3–1.8)	0.56
Time of last abortion					
1 month-1 year	149 (52.3)	96 (66.7)	1.0	1.0	
1–2 years	69 (24.2)	23 (16.0)	0.5 (0.3–0.8)	0.2 (0.1–0.5)	< 0.001
2–3 years	32 (11.2)	9 (6.2)	0.4 (0.2–0.9)	0.1 (0.05–0.4)	< 0.001
Above 3 years	35 (12.3)	16 (11.1)	0.7 (0.3–1.3)	0.4 (0.2–0.9)	0.039

## Discussion

Repeat induced abortion, or having more than one pregnancy termination, is bound in a vicious cycle with repeat unintended pregnancy. Women who have had a recent abortion are more likely to discontinue contraceptive use during a 1-year follow up period and both recent and other previous abortion clients have been found to be more likely to have a (repeat) unintended pregnancy during that time period [19].

This study revealed that the magnitude of repeat induced abortion among abortion care service seekers at Marie Stopes International Ethiopia clinics was 33.6%. This is similar with study done in Ethiopia with the magnitude of 30%. It is consistent with the facility based cross sectional study from Nepal with the magnitude of repeat induced abortion of 32.5% among the reproductive age group of women who seeks abortion care [14, 19, 20].

Also it is similar with study done both in developed and developing countries. For instance, this finding is similar with facility based cross sectional study’s results of Canada (35.5%) [21], Finland (32%) [22], New Zealand (36%) [23] and Sweden (37%) [24]. Also this result is comparable with the findings from developing countries such as facility based study from Kenya (16%) [25], Sudan (40%) [26]. This is may be because of the women in developed and developing regions of the world turn to abortion at similar rates; annually, 29 abortions are performed per 1000 women in developing countries, compared with 26 per 1000 women in developed countries.

Several studies have identified different factors which affect the rate of repeat induced abortion among the reproductive age group of women in both developed and developing countries.

Russian clinic based cross sectional study revealed that low educational status and age below 35 years were the associated factors of repeat induced abortions [10], the present study supports this study findings, here in this study all the participants under the age of 35 years were statistically significantly associated for the repeat induced abortion with different values of (adjusted) odds ratio and *p*-values.

This study showed that age under 35 years, low educational status and middle economic status were the main associated factors of repeat induced abortion among the abortion care seekers, and this finding is supported by the study from Nepal for the factor age and low educational status [14].

A study from Kenya showed that being separated or divorced or widowed and using traditional contraception methods were associated with a higher likelihood of a repeat abortion [25], however, in this study the majority of the participants who had repeat induced abortions were married (58.3%) and single (33.3%) but they are not statistically significant.

This may be because of the fact that, at present study the participants were only taken from the Marie Stopes International Ethiopia clinics which is a profit based non-governmental organization, so this limitation of the sample may not be representative of the whole population as like that of Kenya which was done in five public different hospitals with large sample size.

## Conclusion and recommendation

### Conclusion

The magnitude of repeat induced abortion is similar with the reports from developing countries but it was lower than that of developed countries. Age group (20–24, 25–29 and 30–34 were positively associated with repeat induced abortion), educational level (primary, secondary and collage diploma and above were negatively associated with repeat induced abortion), monthly income (earn 1001–2000 Ethiopian birr were positively where as monthly income between 2001 and 3000 Ethiopian birr negatively associated), years in marriage (1–2 years was negatively associated) and time of last abortion (1-2 years, 2-3 years and above the 3 years were negatively associated) were the associated factors for repeat induced abortion. Health promotion messages are needed to focus to improve the knowledge of women about contraceptives as a primary prevention of repeated induced abortion. .

### Recommendation

Based on the finding of this study the following important recommendations are forwarded for the respective bodies’ such as clinicians, patients, health educators, policy makers, program implementers and researchers who will engage on sexual and reproductive areas as of one main public health concern.

### For organizations and institutions

The ministry of health should have undertake the survey studies either at facility based or community based level to determine the prevalence of repeat induced abortion and its associated factors, Since we did not have the identified prevalence of repeat abortion at national level .

### For health workers

Health promotion messages are needed to focus to improve the knowledge of women about contraceptives as a primary prevention of repeat induced abortion.

### Patients

Awareness creation on the patients on the impact of induced abortion and repeat induced abortion for their future health and IEC for the clients’ towards family planning during post abortion care time.

### For researchers

Finally, it is better to conduct further large scale epidemiological research with large sample size at population level (using both qualitative and quantitative methods).

## Data Availability

The datasets generated and/or analyzed during the current study are not publicly available due to confidentiality. If anyone wants to have a data may contact the corresponding author for data access using the following address: Email: woldemel@gmail.com, P. O.B. 554 and Phone number + 251912097351.

## Abbreviations

ANC: Antenatal Care
AOR: Adjusted Odd Ratio
BSc: Bachelor of Science
CI: Confidence Interval
FP: Family planning
IEC: Information Education Communication
MSIE: Marie Stopes International Ethiopia
PNC: Postnatal Care
